# Incorporation of Nano-Zinc Oxide as a Strategy to Improve the Barrier Properties of Biopolymer–Suberinic Acid Residues Films: A Preliminary Study

**DOI:** 10.3390/ma17153868

**Published:** 2024-08-05

**Authors:** Aleksandra Jeżo, Faksawat Poohphajai, Rene Herrera Diaz, Grzegorz Kowaluk

**Affiliations:** 1 Institute of Wood Sciences and Furniture, Warsaw University of Life Sciences—SGGW, Nowoursynowska St. 159, 02-776 Warsaw, Poland; aleksandra_jezo@sggw.edu.pl; 2 InnoRenew CoE, Livade 6a, 6310 Izola, Slovenia; faksawat.poohphajai@innorenew.eu (F.P.); rene.herdiaz@innorenew.eu (R.H.D.); 3 Faculty of Mathematics, Natural Sciences and Information Technologies, University of Primorska, 6000 Koper, Slovenia; 4 Department of Bioproducts and Biosystems, Aalto University School of Chemical Engineering, 00076 Aalto, Finland

**Keywords:** barrier properties of nanofilms, nano-zinc oxide nanoparticles, suberic acid residues, biodegradable finishing coatings, biological resistance

## Abstract

Finishing coatings in the wood-based composites industry not only influence the final appearance of the product but also serve to protect against fungi and molds and reduce the release of harmful substances, particularly formaldehyde and volatile organic compounds (VOCs). Carbon-rich materials, such as those derived from birch bark extraction, specifically suberin acids, can fulfill this role. Previous research has demonstrated that adding suberin acid residues (SAR) at 20% and 50% by weight significantly enhances the gas barrier properties of surface-finishing materials based on poly(lactide) (PLA) and polycaprolactone (PCL), particularly in terms of total VOC (TVOC) and formaldehyde emissions. This study aims to explore whether these properties can be further improved through the incorporation of nano-zinc oxide (nano-ZnO). Previous research has shown that these nanoparticles possess strong resistance to biological factors and can positively affect the characteristics of nanofilms applied as surface protection. The study employed PLA and PCL finishing layers blended with SAR powder at 10% *w*/*w* and included 2% and 4% nano-zinc oxide nanoparticles. The resulting blends were milled to create a powder, which was subsequently pressed into 1 mm-thick films. These films were then applied to raw particleboard surfaces. TVOC and formaldehyde emission tests were conducted. Additionally, the fungal resistance of the coated surfaces was assessed. The results showed that PLA/SAR and PCL/SAR composites with the addition of nano-zinc oxide nanoparticles exhibited significantly improved barrier properties, offering a promising avenue for developing biodegradable, formaldehyde-free coatings with enhanced features in the furniture industry. Furthermore, by utilizing SAR as a post-extraction residue, this project aligns perfectly with the concept of upcycling.

## 1. Introduction

Surface-finishing technologies in the furniture industry aim to ensure both aesthetic appeal and functional safety, including resistance to hydrolysis [1], biological factors [2], fire retardance [3], and minimizing environmental emissions, depending on the furniture’s intended use. Breakthroughs in green nanotechnology are revolutionizing processes and products, benefiting the environment, reducing pollution, and conserving resources [4,5].

Poly(lactic acid) (PLA) polymers, derived from renewable resources like corn starch or sugarcane [6], offer an eco-friendly alternative for wood-finishing coatings [7], combining sustainability with a durable finish [8,9]. However, due to some drawbacks of PLA, further modifications are implemented, including fiber reinforcement [7,8,9,10,11,12,13,14,15,16], blending [17], or plasticization [18]. The findings by Oksman et al. indicate that PLA serves as an effective matrix material for natural fiber composites, demonstrating promising improvements in the mechanical properties of PLA and flax fiber composites [19].

Aside from PLA, another promising biodegradable polymer is polycaprolactone (PCL), which shows good compatibility with other polymers [20,21,22,23,24,25]. It has also been proven beneficial to incorporate lignocellulosic particles/fibers into PCL to enhance its properties [16,21,26,27] or other compounds like kaolin [28] or bioactive glass [29].

Incorporating nanoparticles into polymers can improve barrier properties due to two main factors: 1. reduction in available diffusion area, achieved by replacing permeable polymer with impermeable nanoparticles; 2. increased path length for the permeant to traverse the film, as it follows a convoluted route around the impermeable nanoparticles [30]. The dispersion of nanoparticles in polymer matrices affects the barrier properties created by making gas fighting more difficult, as the added nanoparticles create a “more difficult”, tortuous gas prevention path [31]. Moreover, the added nanoparticles cause changes in the polymer matrix itself in the interfacial areas. If polymer–nanoparticle interactions are favorable, polymer strands in close proximity to each nanoparticle can be partially immobilized. The result is a reduced velocity of gas particles [32,33]. The incorporation of nanoparticles into polymers, also PLA and PCL, is an area of research that holds promise for enhancing the properties and functionality of these biodegradable materials. Incorporating nanoparticles like SiO_2_, Al_2_O_3_, TiO_2_, and Fe_2_O_3_ into Poli-L-lactide (PLLA) significantly improves the barrier properties of the resulting nanocomposites, reducing water vapor permeability by up to 18% and wet oxygen permeability by up to 9% [34]. By applying CaCO_3_ nanoparticles up to 5 wt% the gas permeability, the results of N_2_, O_2_, and CO_2_ revealed an improvement in the final barrier properties [35].

Inorganic metal oxide nanoparticles (e.g., TiO_2_, ZnO, SiO_2_, and Al_2_O_3_) can also absorb UV radiation; however, due to naturally wide bond gaps, their UV absorption is not complete. Nano-ZnO deposited on wood by Kong et al. [36], forming dense and uniform arrays of nanorods, provided UV protection, improved photostability, enhanced flame retardancy, and water repellency when modified with stearic acid. Regarding the organic compounds, cellulose nanocrystals (CNCs) and lignin nanoparticles (LNPs) enabled the creation of PVC-based composites with improved barrier properties, limiting gas migration and enhancing the UV-shielding [37].

**Figure 1:**
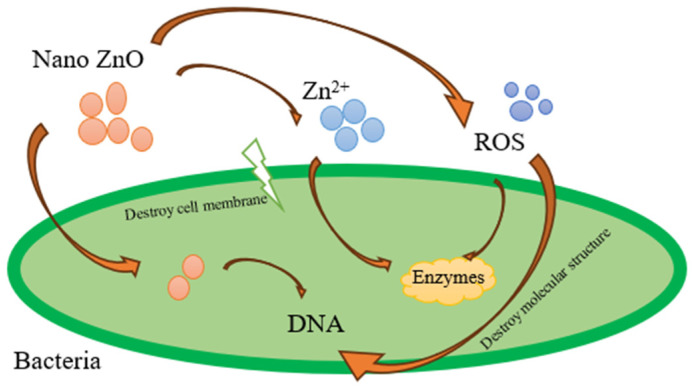
Antibacterial mechanism of ZnO NPs (own elaboration based on [46]).

One of the other potential improvements that the implementation of nanoparticles can bring is making the composite bioprotective. Nanomaterials have recently become important in many industrial paths due to the larger percentage of atoms at their surface, which lead to high surface reactivity. ZnO nanoparticles have excellent antibacterial properties since they are of a reduced size. Direct contact of nano-ZnO leads to the destruction of bacterial cell integrity [38]. Once the nanoparticles are inside the bacterial cell, they generate ROS (reactive oxygen species), and release of antimicrobial ions, mainly Zn^2+^ [39]. ZnO possesses significant photocatalytic efficiency and is more biocompatible than TiO_2_ [40]. Nano-ZnO in aqueous solution under UV radiation presents a phototoxic effect that leads to producing ROS-like hydrogen peroxide (H_2_O_2_) and superoxide ions (O_2_^−^). The generated active species penetrate the cells, inhibiting or killing microorganisms. Figure 1 presents a schematic of the mechanism of antibacterial the working of nano-ZnO. It has also been proven that the addition of metal-based particles, such as copper [41,42] and silver [15,43,44], into polymers is a versatile route to take advantage of their strong antimicrobial properties, producing novel biocide materials [45]. Moreover, as Wu et al. found out in the example of organoclays, the introduction of nanofillers leads to a significant enhancement in phase morphology, primarily due to their strategic localization, which effectively prevents the merging of discrete domains and encourages the breakup of droplets [25].

Carbon-rich natural substances, like those found in birch bark extraction products, such as suberic acids (SAs) and their residues (SARs), have significant potential for reducing the emissions of harmful substances into the environment. The findings of Jeżo and Kowaluk [16] indicate a positive influence of a 20% and 50% addition of SAR into PLA and PCL blends regarding TVOC and formaldehyde emission from plywood. This can be attributed to the high lignin content in SAR particles. Furthermore, the SAR addition enhances the relative hardness and scratch resistance of PLA- and PCL-based surface-finishing materials. This paper investigates whether adding nano-zinc oxide (nano-ZnO) can improve the barrier properties and biological resistance of nanocomposites created by blending PLA/PCL, SAR, and nanoparticles. While it has been shown that the addition of nano-ZnO to PLA/PCL has potential in medical applications [47,48], it remains to be found if such potential also lies in the wood composites industry. Both the effects of SAR addition and nanoparticle incorporation will be investigated.

## 2. Materials and Methods

### 2.1. Materials

The surface-finishing layers were fabricated using two distinct matrices: laboratory-purpose PLA (Sigma-Aldrich, Saint Louis, MO, USA, product no. 38534) and PCL (Sigma-Aldrich, product no. 704105) in drops with a diameter of 3 mm. The suberinic acid residues (SAR) used in the research, which were utilized to prepare the blends, have been described in detail by Makars et al. [49]. The following are the basic chemical properties of SAR: cellulose 9.0 wt%, aromatic suberin, lignin 21.4 wt%, ω-hydroxy acids 17.5%, and α, ω-diacids 11.9%. The nanoparticles used in the study were nano-zinc oxide (nano-ZnO), hereafter called “nano”, obtained from ASTON Chemicals, Warsaw, Poland, with a diameter of 265 nm. Methylene chloride (CH_2_Cl_2_) (Sigma-Aldrich, product no. 34856), toluene (C_6_H_5_CH_3_) (Sigma-Aldrich, product no. 320552), and Potato Dextrose Agar (Sigma Aldrich, product no. 70139).

### 2.2. Blends Processing Methods

(1) The PLA finishing layer was made by mixing methylene chloride (CH_2_Cl_2_) solution for PLA, 21% dry matter content, with SAR powder, 10% *w*/*w*, respectively, hereafter called “PLA/SAR”; and with nano-ZnO particles, 2% and 4%, *w*/*w*, respectively, hereafter called “PLA/SAR nano 2%” and “PLA/SAR nano 4%”. The samples with the addition of nano-ZnO but without SAR are hereafter called “PLA nano 2%” and “PLA nano 4%”. A pure PLA surface-finishing layer has also been tested (hereafter called “PLA”) as a reference. All the prepared blends were spread on Polytetrafluoroethylene (PTFE) sheets under a fume hood to evaporate the solvent and then milled to attain a powder size smaller than 0.1 mm. Such a powder was formed in a hot press (as mentioned above), as described by Gumowska et al. [50]. The prepared film was pressed in a hot press (pressing time, 75 s; temperature, 185 °C; and pressure, 0.8 MPa) onto the unfinished particleboard surface described above.
(2) The PCL finishing layer was obtained from toluene (C_6_H_5_CH_3_) solution for PCL, 25% dry matter content. The remaining steps of the surface-finishing preparation are the same as those described above for PLA. Using this method, the subsequent samples were attained: PCL (hereafter called “PCL”; no SAR/nano-ZnO addition, reference sample) “PCL/SAR”, “PCL/SAR nano 2%”, “PCL/SAR nano 4%”, “PCL nano 2%”, “PCL nano 4%”.

According to the research plan, the surface-finishing layers created on the particleboard base were exposed to ambient conditions (20 °C; 65% R.H.) until a constant weight was achieved over a span of seven days before tests. All the variants and their composition were presented in Table 1.

From each variant, one sample was grinded and pressed on the surface twice, to investigate the effect of repeated processing on the relative hardness.

### 2.3. Relative Hardness

Relative hardness was measured on a pendulum apparatus type AWS—9 (POL-ZAF S.C., Wrocław, Poland) according to the procedure described in the ISO 1522 standard [51]. As many as 2 repetitions per tested sample type were performed.

### 2.4. Cold Liquids Resistance

Cold liquid resistance was measured according to the procedure described in the EN 12720+A1 standard [52]. As many as 2 repetitions per tested sample type were performed. The following cold liquids were employed: acetone, citric acid, ethanol, and distilled water. The exposition time equaled 24 h.

### 2.5. Total VOC and Formaldehyde Emission

The total VOC and formaldehyde emission tests were completed in an emission test chamber at a temperature of 23 °C +/− 0.5 °C and relative air humidity of 44% +/− 1%. Other parameters of sample conditioning were performed following the EN 717-1 standard [53]. The TVOC and formaldehyde emission tests were carried out after conditioning the samples for 24 h by analyzing the chamber air over the three repetitions after 20 min each using a JD-3002 Air Quality Tester (Dongguan Jinlide Electronic Technology Co., Ltd., Dongguan City, Guangdong Province, China). We adhered to the standards regarding air-conditioning conditions for samples during the emission test.

### 2.6. Antifungal Test

In the antifungal study, the disk diffusion method was employed to assess the growth inhibition of the materials on specific mold fungi. A 90 mm Petri dish containing a Potato Dextrose Agar was centrally inoculated with a 100 µL spore suspension of two common mold species, namely *Aspergillus niger* and *Cladosporium cladosporioides*. The spores were evenly spread using a sterile plastic spreader. Subsequently, one test disk of each type of test material was placed in the center of the plate, and three replicas of each material were employed for each mold species. The plates were incubated in a growth chamber in the dark under a control temperature of 25 °C. After 10 days of incubation, the growth of fungi was visually evaluated using a Keyence VHX-6000 digital microscope (Keyence, Osaka, Japan). The growth of mold was assessed based on the percentage of mold coverage on the surface. To ensure uniform evaluation of the test, the following six-grade assessment scheme was applied in this experiment:

Grade 0 = no infestation of the surface.
Grade 1 = 1–20% infestation of the surface.
Grade 2 = 21–40% infestation of the surface.
Grade 3 = 1–60% infestation of the surface.
Grade 4 = 61–80% infestation of the surface.
Grade 5 = 81–100% infestation of the surface.

### 2.7. The Effect of Repeated Processing of Polymer Blends on Their Hardness

The samples were pressed onto the board surface, as described in Section 2.1. and subsequently recovered from the surfaces by scratching off. Then, the recovered blends were ground and pressed onto the surfaces again. One sample of each type was processed this way, and then the relative hardness was examined as described in Section 2.2.

### 2.8. Statistical Analysis

Analysis of variance (ANOVA) and *t*-test calculations were used to verify significant differences (α = 0.05) between factors and levels where appropriate, using the RStudio software 2024.04.2 Build 764 © 2009–2024 Posit Software, PBC (R Foundation, Vienna, Austria).

## 3. Results and Discussion

### 3.1. Cold Liquids Resistance

The cold liquids resistance evaluation results of the tested surface-finishing layers are presented in Table 2. When it comes to pure PLA, only in the case of citric acid and distilled water was slight cloudiness found. There was no significant influence of the addition of SAR to PLA regarding the resistance to cold liquids. The pure PCL layer was more resistant when referred to PLA. No changes in the surface were found. The addition of SAR to PCL did not significantly lead to the lowering of the resistance of the tested blends. Only in the case of PCL/SAR nano 4% sample was there a slight change observed when affected by ethanol. To compare the results with the commercial coatings, let us regard waterborne coatings (acrylate-based and polyurethane-based). Pavlic et al. [54] found that the best resistance to cold liquids occurred in the case of exposure to water and citric acid, while the fastest changes were observed after acetone application. The coatings that were acrylic-based showed better resistance compared to the polyurethane-based ones. It was also stated by Lis et al. [55] that most of the agents had caused no changes in waterborne UV acrylic coatings even after 24 h. Only the ethyl alcohol showed negative impact on evaluated surfaces, causing slight changes.

### 3.2. Total VOC and Formaldehyde Emission

Table 3 shows the results of testing the emission of free formaldehyde (HCHO) and (VOCs) in samples covered with coatings on both sides. A particleboard not covered with any coating was used as a control sample (REF). There was a statistically significant reduction in TVOC and HCHO emission between all of the variants tested when the investigated surface-finishing layers were applied. The addition of SAR reduces the revealed emissions. However, the lowest emissions values were obtained for the samples with nano-ZnO incorporated, with the PCL nano 2% and 4% performing the strongest gas barrier properties. The polymer matrix was the only significant factor in this test. The mentioned reduction in TVOC and formaldehyde emission from the particleboard covered with different surface-finishing layers can be an effect of the scavenger nature of lignin, which is the main component (over 21 wt%) of SAR. The SAR-containing surface-finishing layers act as a bi-functional barrier layer, which can avoid the gas transfer from the core of the composite to the environment, as well as can fix the formaldehyde and other emitted compounds in the finishing layer structure [56]. According to [57,58], the blends of PLA, as well as PCL, can be recognized as extremely promising for the development of bio-based and biodegradable polymeric materials with low oxygen permeation, that is, for the development of suitable alternatives to conventional and highly pollutant oil-based plastics. It can be concluded that the tested surface-finishing materials, based on biopolymers with SAR incorporation, have promising features regarding the gas barrier layer on wood-based material surfaces. It is moreover notable that nanoparticles exhibit great advantages since they can reduce formaldehyde emissions through indirectly increasing the curing degree of the resins, thus resulting in improved hydrolytic stability, due to the increased chemical bonds of the resin components with free formaldehyde. Furthermore, the reaction of hydroxyl groups occur on the nanomaterial surface with free formaldehyde, and such coatings are able to perform barrier properties or a “shielding effect” [59]. As [60] found, the application of 8% zinc results in a reduction in formaldehyde emission by an average of 44%.

### 3.3. Relative Hardness

**Figure 2:**
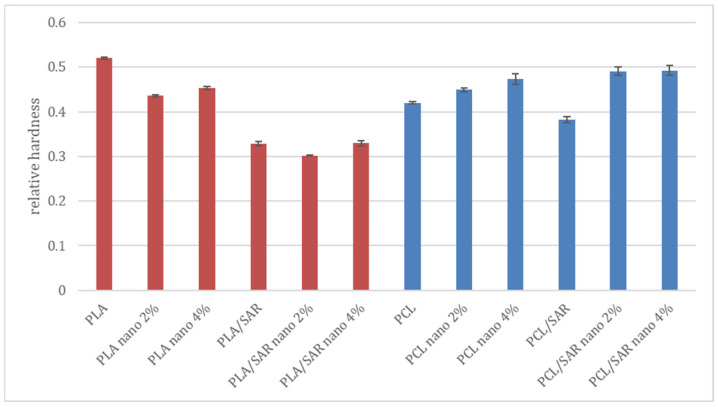
The results of the relative hardness of the examined coatings (red—PLA and PLA blends; blue—PCL and PCL blends).

Figure 2 shows the results of testing the relative hardness of the produced coatings. In coatings made on a PLA matrix, the sample made of pure polymer without additives (0.52) showed the highest hardness. For the sample with a 2% addition of nano-ZnO, a decrease in hardness to 0.43 was observed; meanwhile, when the share of nanoparticles was increased, there was a slight increase in hardness (0.45). The PLA-based samples containing SAR showed statistically significantly lower hardness: 0.33, 0.30, and 0.33 for the PLA/SAR variants, PLA/SAR nano 2%, and PLA/SAR nano 4%, respectively. As for the samples produced based on the PCL matrix, the pure polymer showed a hardness of 0.42. The nano-ZnO tax resulted in an increase in hardness to 0.45 and 0.47, respectively. For the PCL/SAR sample, a decrease in hardness to 0.39 was observed, while for the PCL/SAR nano 2% and PCL/SAR nano 4% variants, an increase in hardness to 0.50 and 0.48 was observed, respectively. Statistical analysis showed that in the tested samples, statistically significant differences were related to the addition of SAR, the type of matrix, and the interaction between the matrix and SAR. The addition of nanoparticles did not have a statistically significant impact on the obtained results.

Previous research conducted by Jeżo and Kowaluk [16] on PLA and PCL blends with SAR showed that the addition of 20% and 50% SAR causes an increase in hardness. However, ref. [61] stated in their study that the mechanical properties of a PLA/IFR (intumescent flame retardant (IFR)-graphene oxide (GO)) blend when incorporated with nano-ZnO were diminished seriously; the tensile strength decreased by 67%, the impact strength decreased by 69%, and the elongation at break was off by 70%. The authors explain their findings with a nano-ZnO-catalyzing PLA, which decomposed into small chains with low molecular and then was destroyed easily. Also, [62] stated that the addition of ZnO nanoparticles introduced an accelerated degradation of the PLA/PCL blends. Rheological results showed that with increasing nanoparticles, the elastic modulus and the complex viscosity decreased, which correlated with the degradation and chain scission of polymeric chains induced by nano-ZnO. The mentioned references are applicable since an approximately linear correlation between the hardness and elastic modulus was established [63]. Moreover, the tensile strength and the hardness of a material are highly correlated [64]. The large size of the nanoparticles could also have influenced the negative results. As shown by [65], big size and aggregates/agglomerates weaken the positive attributes of nanoparticles in nanocomposites. Small nanoparticles and a thick interphase present high levels for the B parameter (interfacial parameter which shows the level of interfacial adhesion), tensile strength, interphase volume fraction, and interphase parameter. Ref. [66] confirms that by decreasing the particle size from 300 nm to 10 nm, the SiO_2_ nanoparticles become drastically harder (∼39×), stiffer (∼15×), and tougher (>3.5×).

Since the SAR showed statistical significance for the obtained results, the decrease in hardness of samples containing PCL and PLA can also be attributed to its addition. Because improved mechanical properties, including hardness, were observed in the literature after adding natural fibers to polymers [16,60], the authors intend to take a closer look at the observed phenomenon and link it with manufacturing parameters and further processing. The worsening of the relative hardness because of the incorporation of the SAR can be due to its alkaline form. It has been confirmed by [67] that alkali treatment can cause a depolymerization of polymers. Also, [68] reported that at a lignin content of 10 wt%, heterogeneous morphology was observed, suggesting phase separation between PLA and alkali lignin. Similar observations were shown in PLA/kraft lignin blends with lignin concentrations higher than 10 wt% [69,70]. This heterogeneity might be an indication of the weak mechanical properties of the investigated system at concentrations higher than 10 wt%.

Comparing the obtained results with different polymer matrixes, it is worth mentioning that by increasing nano-ZnO up to 0.3% in a polystyrene polymer matrix, there is a remarkable increment of approximately 47% in the hardness [71], while the hardness can be decreased by 5.73% when added into natural rubber [72]. Incorporation of ZnO–Ag hybrid nanoparticles into the acrylate epoxy matrix increases its pendulum hardness [73]; meanwhile, compared with neat polyurethane acrylate (PUA), the hardness in films increases from 0.03 to 0.05 GPa when ZnO in a share of 5% is incorporated [74].

Degradation causes changes in the mechanical properties, particularly when the molecular weight importantly decreases. Reinforcements could attenuate the loss in mechanical properties, depending on its structural integrity and the state of interfacial adhesion [75].

### 3.4. Antifungal Test

**Figure 3:**
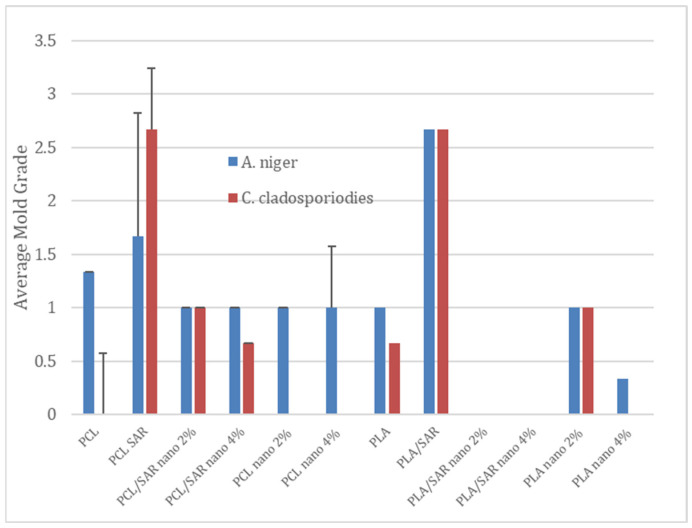
The average mold grade of the examined coatings exposed to *A. niger* and *C. cladosporioides*.

**Figure 4:**
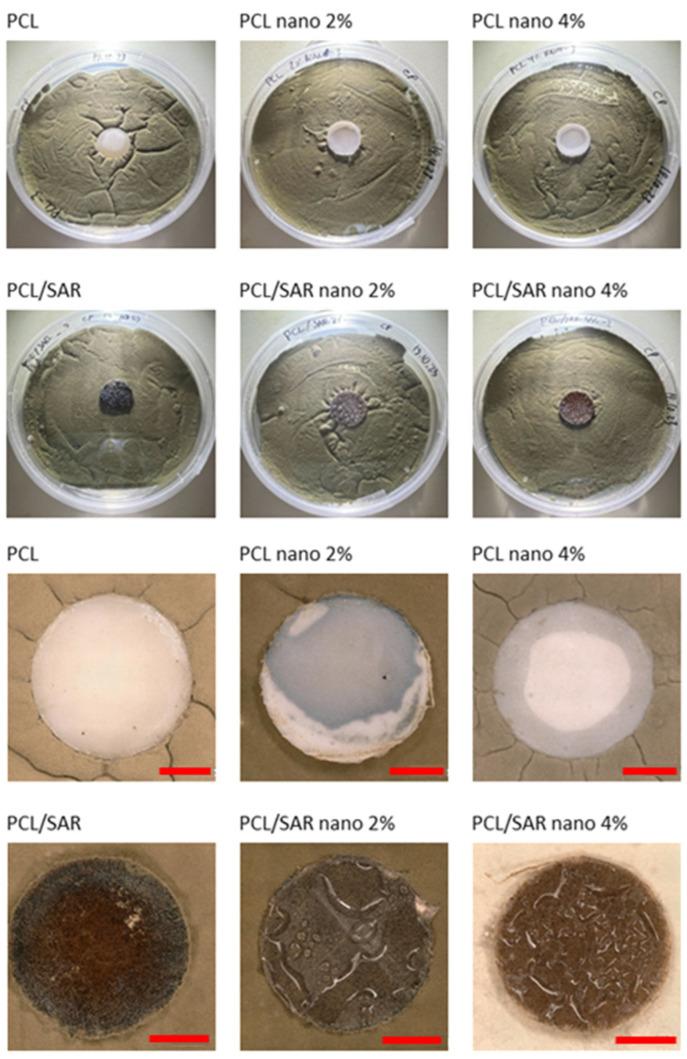
PCL tested with *Cladosporium cladosporiodies* (scale bar: 3 mm).

**Figure 5:**
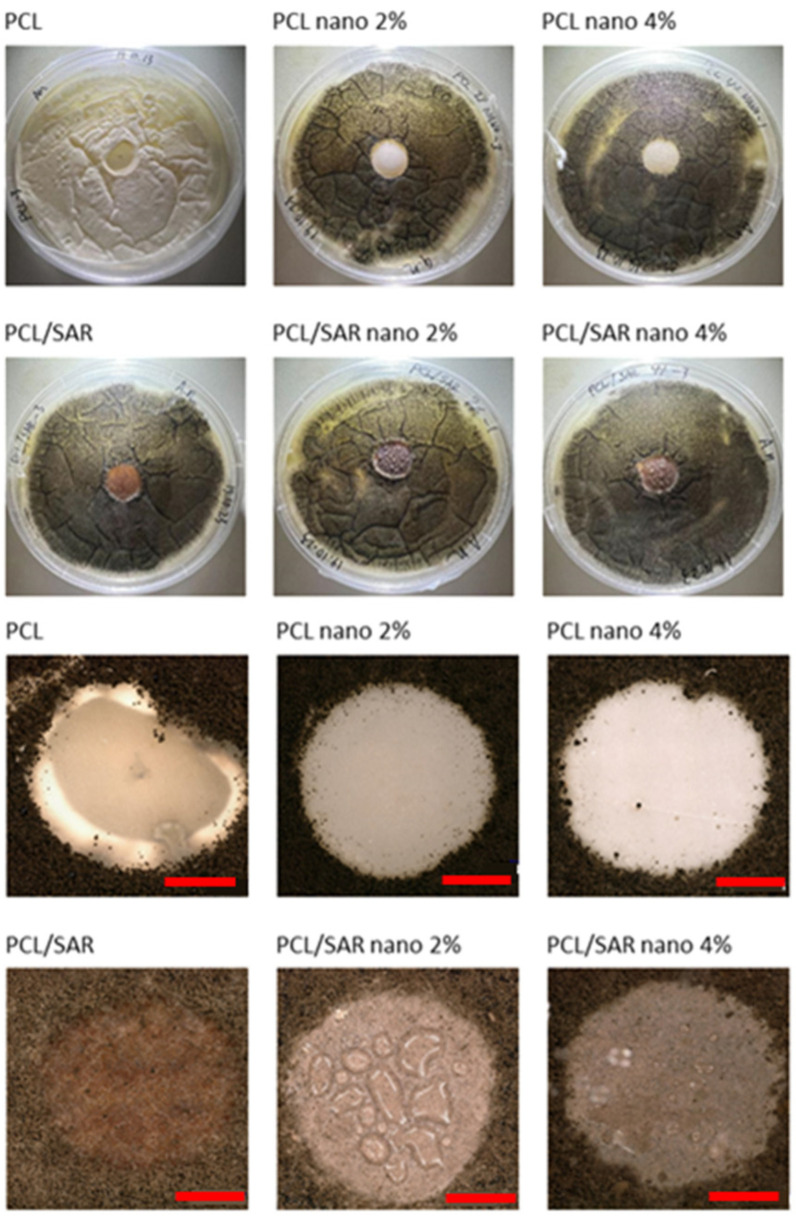
PCL tested with *Aspergillus niger* (scale bar: 3 mm).

**Figure 6:**
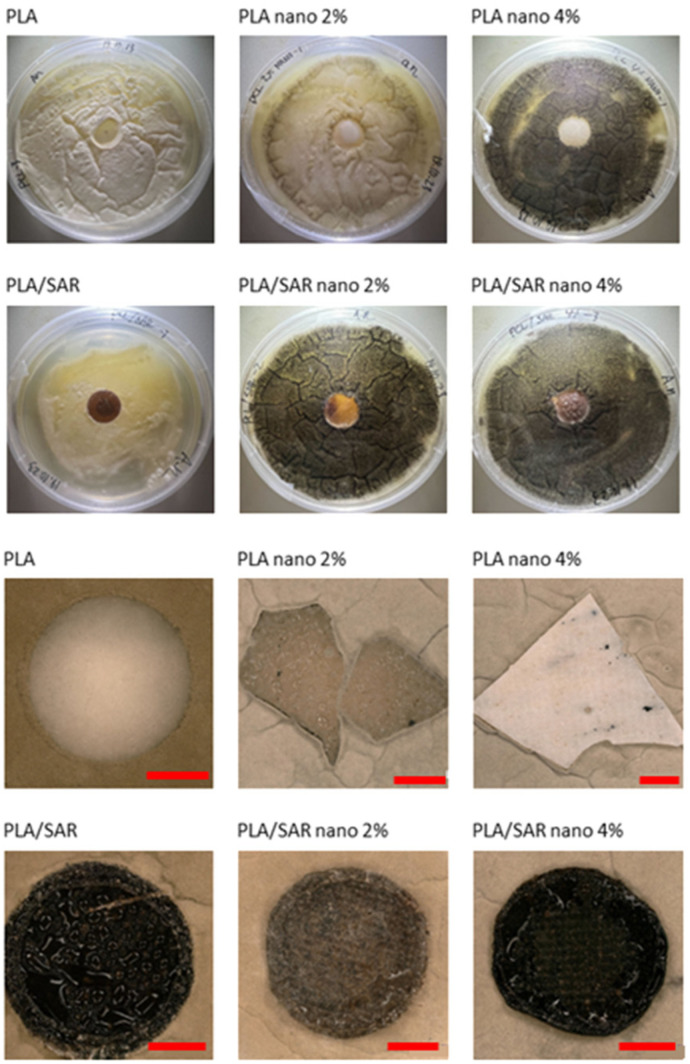
PLA tested with *Cladosporium cladosporiodies* (scale bar: 3 mm).

**Figure 7:**
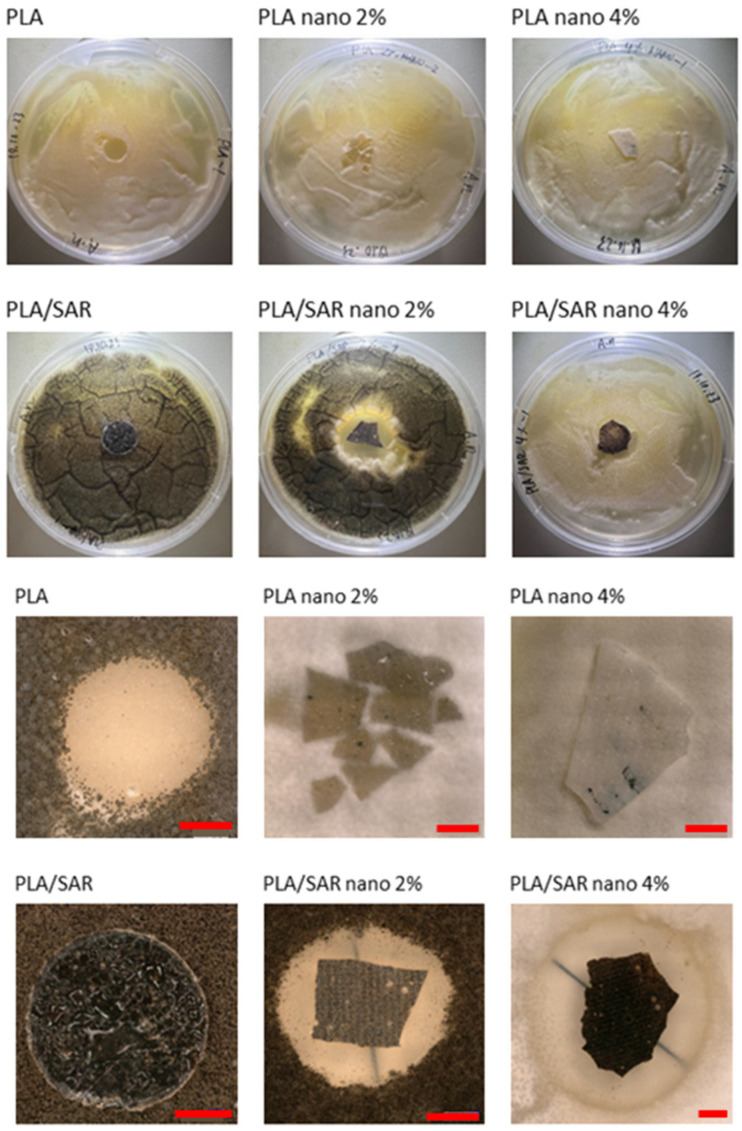
PLA tested with *Aspergillus niger* (scale bar: 3 mm).

In Figure 3, the results of the antifungal tests are illustrated, with a demonstration of average mold grade values. In Figure 4, Figure 5, Figure 6 and Figure 7, the photos of the infected samples are shown. In the case of samples containing PLA and additives, specifically PLA/SAR nano 2%, PLA/SAR nano 4%, and PLA nano 4%, a higher antifungal effect was observed, with no contact between the fungal spores and the material, in contrast to the PLA control. Additionally, PLA/SAR nano 2% and PLA/SAR nano 4% demonstrated an inhibition zone against *A. niger*, indicating their ability to prevent or impede fungal growth. Similar antifungal effects were observed in PLA samples against *C. cladosporioides*, with the highest activity in the PLA/SAR nano 2%, PLA/SAR nano 4%, and PLA nano 4% samples. An exception was found in the PLA/SAR samples, which proved to be the most susceptible to *A. niger* and *C. cladosporioides*. Conversely, the samples containing PCL and additives generally exhibited better performance, particularly against *C. cladosporioides*, notably in the PCL/SAR nano 4%, PCL nano 2%, and PCL nano 4% samples. However, even the PCL control showed some inhibitory effect against *C. cladosporioides*. In the case of *A. niger*, the PCL samples displayed similar performance in PCL/SAR nano 2%, PCL/SAR nano 4%, and PCL nano 2%, where few spores were observed, unlike the control sample (PCL) and PCL/SAR, where the samples were colonized by *A. niger*. The statistical analysis revealed the significant impact of nanoparticles, type of matrix, SAR added, as well as the correlation between SAR and nano-ZnO on the mold grade.

As other studies confirm, the silane-modified ZnO-hybrid-embedded Poly(methyl methacrylate) (PMMA) offers resistance against fungal growth when infected with *Aspergillus niger* and *Aspergillus flavus* [76]. Nano-ZnO shows antifungal activity against *Aspergillus niger* with an MIC of 2.5 mg/mL. Thus, nano-ZnO is twice as potent in killing *Aspergillus*, as compared to its non-nano-counterpart (micro ZnO) [77].

### 3.5. Relative Hardness after Repeated Processing of the Samples

**Figure 8:**
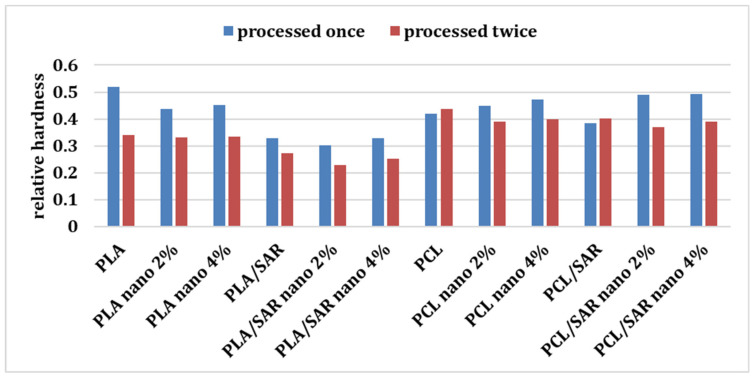
The relative hardness of the samples after one and after repeated processing.

Figure 8 shows a comparison of the relative hardness of the tested coatings ground and pressed once and twice. A decrease in the value of the tested parameter (Table 4) can be noticed for samples whose coating was reprocessed after ironing. The decrease in hardness recorded for the samples is shown in the table. Greater differences were observed in the case of samples made based on the PLA matrix than the PCL matrix. Due to the re-processing of the PLA-based blends, the polymer degraded, significantly affecting its mechanical properties, like the loss of hardness. Ref. [78] reports similar observations for PLA blends with starch, where the degradation of PLA was confirmed by SEM tests. The authors observed a decrease in the flexural modulus and impact strength values due to repeated processing of the blends. The degradation of PLA due to repeated processing is also confirmed by [79], who investigated the impact of repeated extrusion on the properties of PLA/HDPE and PLA/PC blends. As demonstrated by [80], mechanically recycled PLA has lower mechanical properties compared to virgin PLA, with impact strength and hardness decreasing by 50% and 4%, respectively.

Although the literature lack examples of a decrease in hardness after regrinding for PCL, researchers have considered many other polymers—polyoxymethylene (POM) [81], thermoplastic polyurethane elastomers [82], or polypropylene [83]—which may confirm a similar trend for PCL as demonstrated in this study. However, polymers have been found that increased their hardness after couple processing cycles, such as high-impact polystyrene [84].

The statistical analysis showed statistically significant differences between the values obtained for hardness after the first and second processing cycle, which confirms that repeated grinding and pressing of polymer blends statistically reduces their relative hardness. Moreover, the only factor that had a statistically significant impact on the percentage decrease in hardness after reprocessing was the type of polymer matrix.

## 4. Conclusions

The following study aimed to investigate some preliminary examinations on the surface-finishing coatings containing suberinic acid residues (SAR) with nano-zinc oxide (nano-ZnO). The tests included resistance to cold liquids, relative hardness, formaldehyde and VOC emissions and antifungal tests. The results revealed no worsening when exposed to cold liquids in the case of samples based on the PCL matrix, with slight changes in some samples based on the PLA polymer. The relative hardness tests showed a decrease in hardness regarding PLA-based samples when incorporated with nanoparticles, and the PCL-based samples presented a decrease in hardness in the case of SAR incorporation. This phenomenon needs to be further investigated; however, the authors suggest that the reason for this worsening might have been the alkali character of the SAR-incorporated sample. It was concluded that the incorporation of SAR and nano-ZnO enabled the minimization of both the emissions of free formaldehyde as well as TVOC. The investigated samples with fillers incorporated exhibited moreover good antimicrobial properties. Moreover, the impact of repeated processing on the relative hardness was regarded, leading to an observation of a decrease in this parameter for both the PLA and PCL. The authors see great potential in the findings obtained, and the topic will be continuously investigated.

## Figures and Tables

**Table 1:** The composition of tested surface-finishing layers.

Variant Label	Matrix	SAR Filler Content (*w*/*w* of Dry Matter)	Nano-ZnO Content (*w*/*w* of Dry Matter)
PLA	PLA	0	0
PLA nano 2%		0	2
PLA nano 4%		0	4
PLA/SAR		10	0
PLA/SAR nano 2%		10	2
PLA/SAR nano 4%		10	4
PCL	PCL	0	0
PCL nano 2%		0	2
PCL nano 4%		0	4
PCL/SAR		10	0
PCL/SAR nano 2%		10	2
PCL/SAR nano 4%		10	4

**Table 2:** Resistance to cold liquids of the tested finishing layers.

Variant Label	Acetone	Citric Acid	Ethanol	Distilled Water
	mg m^−3^			
PLA	A	B	A	B
PLA nano 2%	B	C	C	B
PLA nano 4%	C	C	B	A
PLA/SAR	B	B	A	A
PLA/SAR nano 2%	C	C	B	C
PLA/SAR nano 4%	B	B	C	C
PCL	A	A	A	A
PCL nano 2%	A	A	A	A
PCL nano 4%	A	A	B	A
PCL/SAR	A	A	A	A
PCL/SAR nano 2%	A	A	A	A
PCL/SAR nano 4%	A	A	A	A

**Table 3:** The TVOC and HCHO emissions of the tested finishing layers.

Variant Label	TVOC	HCHO
	mg m^−3^	
REF	0.245	0.044
PLA	0.220	0.039
PLA nano 2%	0.172	0.030
PLA nano 4%	0.179	0.031
PLA/SAR	0.222	0.041
PLA/SAR nano 2%	0.218	0.039
PLA/SAR nano 4%	0.216	0.040
PCL	0.264	0.045
PCL nano 2%	0.068	0.012
PCL nano 4%	0.065	0.012
PCL/SAR	0.080	0.014
PCL/SAR nano 2%	0.078	0.013
PCL/SAR nano 4%	0.077	0.016

**Table 4:** Percentage decrease in the relative hardness of the examined samples after the second processing.

Variant Label	Hardness Decreases after the Second Pressing
PLA	34.60%
PLA nano 2%	23.72%
PLA nano 4%	26.14%
PLA/SAR	16.57%
PLA/SAR nano 2%	24.62%
PLA/SAR nano 4%	23.32%
PCL	−3.81%
PCL nano 2%	13.28%
PCL nano 4%	15.49%
PCL/SAR	−5.00%
PCL/SAR nano 2%	24.66%
PCL/SAR nano 4%	20.73%

## Data Availability

The data presented in this study are available on request from the corresponding author. The data are not publicly available due to sensitive nature of the research. The participants of this study did not give written consent for their data to be shared publicly (commercial reasons, ongoing research etc.).
